# Designing a Scoring System for Differential Diagnosis From Reactive Thrombocytosis and Essential Thrombocytosis

**DOI:** 10.3389/fmed.2021.736150

**Published:** 2021-12-16

**Authors:** Chih-Lung Shen, Tsung-Cheng Hsieh, Tso-Fu Wang, Wei-Han Huang, Sung-Chao Chu, Yi-Feng Wu

**Affiliations:** ^1^Department of Hematology and Oncology, Hualien Tzu Chi Hospital, Buddhist Tzu Chi Medical Foundation, Hualien, Taiwan; ^2^Institute of Medical Sciences, Tzu Chi University, Hualien, Taiwan; ^3^College of Medicine, Tzu-Chi University, Hualien, Taiwan; ^4^Department of Clinical Pathology, Hualien Tzu Chi Hospital, Buddhist Tzu Chi Medical Foundation, Hualien, Taiwan; ^5^Ph.D. Program in Pharmacology and Toxicology, Tzu Chi University, Hualien, Taiwan

**Keywords:** scoring system, reactive thrombocytosis, essential thrombocytosis, white blood count, hemoglobin, platelet count, mean platelet volume

## Abstract

**Background:** Thrombocytosis is a common finding in hospitalized patients and is of two main types, essential thrombocytosis (ET) and reactive thrombocytosis (RT). It is important to distinguish the two due to increased risk of developing marrow fibrosis, acute leukemia, and thrombosis in the former. Molecular studies are the main tools to differentiate the two but are not available in all hospitals. We aimed to design a highly sensitive scoring system using routine lab data to classify thrombocytosis as essential or reactive.

**Methods:** A total of 145 patients were enrolled in this study. Potential predictors included patient demographics and clinical laboratory parameters. Receiver operating characteristic curve analysis was used to decide the optimal cutoff level. Multivariate logistic regression with forward model selection method was performed to decide the predictors.

**Results:** The risk scores by multivariate analysis were as follows: 1 point for WBC > 13,500/μL; 2.5 points for Hb > 10.9 g/dL; 3 points for platelet count > 659,000/μL; and 2 points for MPV > 9.3 fL. The cut off value was set as 4.5 points, and sensitivity of 91.1% and specificity of 75.8% were noted.

**Conclusion:** In this study, we investigated lab data and developed a high-sensitivity convenient-to-use scoring system to differentiate ET from RT. The scoring system was assigned to the resulting model to make it more economical, simple, and convenient for clinical practice.

## Introduction

Platelets play an important role in bleeding and blood clotting. The normal platelet count in adults ranges from 150,000 to 450,000 per microliter. A condition in which the platelet count exceeds 450,000/μL is defined as thrombocytosis. Thrombocytosis is a common finding in hospitalized patients and is reported in patients admitted for trauma (1), intensive care (2), and treatment of cancer (3). By Kim et al.'s reports, inpatient consultations to hematologists have increased in incidence. Under the 354 hematologists' consultations with abnormal hematologic manifestation, anemia, thrombocytopenia, and pancytopenia were the major problems, but thrombocytosis was also noted in 7% of all patients hospitalized require hematology consultations (4). Thrombocytosis can be classified into two types, essential (primary) thrombocytosis and reactive (secondary) thrombocytosis, and distinction between the two is important for evaluation, prognosis, and treatment (5).

Studies have reported that 80 to 90% of the cases are diagnosed with reactive thrombocytosis (5). Reactive thrombocytosis is defined as high platelet count due to underlying disease or that caused by medications. The causes include acute or chronic blood loss, iron deficiency, asplenia, malignancy, chronic inflammatory conditions, and infectious diseases (5). In contrast, essential thrombocytosis is associated with the myeloproliferative neoplasms and results from unregulated abnormal platelet production from the bone marrow progenitor cells. The abnormal proliferation of mature bone marrow cells is observed in all three lineages: leukocytes, erythrocytes, and megakaryocytes (6).

Extreme thrombocytosis may induce thrombotic events such as acute myocardial infarction, deep vein thrombosis, and pulmonary embolism. Although the platelet count in reactive thrombocytosis can exceed 1 × 10^12^ cells/liter, thrombovascular complications are rare. In contrast, more thrombotic events are reported with essential thrombocytosis (6). Furthermore, about 3.9% of patients with ET will develop marrow fibrosis, and 2.8% of patients with ET will transfer to acute leukemia. The incidences are much higher than the patients with RT (7). Therefore, distinguishing between the two types is essential for clinical practice.

Serum chemistry tests, such as serum ferritin and C-reactive protein levels, can assist in distinguishing RT from ET (6). However, these tests do not provide consistent results. Since the discovery of a phenotypic driver mutation JAK2V617F in essential thrombocytosis more than a decade ago, additional gene mutations, including in CALR, MPL, and CBL, have been identified (8–10). Although a diagnostic workup with an emphasis on molecular studies is currently recommended by the World Health Organization (WHO) (11), over 10% patients do not exhibit the aforementioned molecular mutations still (12). The exclusion of secondary causes is still necessary in patients with suspected ET who lack these molecular mutations. Furthermore, there is limited access to molecular studies, as these are not available in all hospitals; yet another drawback is that while these tests are accurate, they are also expensive and require several days to weeks to provide results.

It is important to distinguish RT from ET, so we aimed to design a scoring system with high sensitivity, using just one routine test with complete blood counts, to assist with this. We hope to design a scoring system for the doctors of family medicine or general practitioners and help them to identify possible bone marrow disease and consult hematologists. They could have hints to transfer the patients to hematologists for arranging further bone marrow examination and molecular studies, which may be not available in hospital or local clinics. This scoring system was assigned to the resulting model to make it more economical, concise, and convenient for use in clinical practice.

## Patients And Methods

### Patients and Data Collection

We collected the patients from our hospital, including inpatients and outpatients. The patients who were admitted to the hematologic ward, visited the hematologic outpatient department, or consulted hematologists by primary survey physicians or teams, were enrolled in this study. Because of hematologists' visits, we could follow on these patients who were diagnosed with RT or ET finally by further laboratory data or treatment. If the patients did not refer to or visit the hematologic department, they had been excluded from this study. Because of above, all the patients had the study of JAK2V617F. Between January 1st, 2010, and June 30th, 2019, a total of 145 patients were enrolled in this retrospective study. All patients had high platelet counts, more than 450,000 per microliter. The patients in whom the platelet counts recovered to normal (<450,000 per microliter) without JAK2V617F mutation and cytoreductive therapy during follow-up were identified as RT. ET was diagnosed based on the presence of any one of the following criteria: (1) JAK2V617F mutation, (2) CALR mutation, (3) bone marrow examination showing results typical for ET such as a hypercellular bone marrow, increased numbers of megakaryocytes, giant megakaryocytes, clusters of megakaryocytes, or other dysplastic megakaryocytic changes, as per the 2016 WHO guidelines (13). In our hospital, because the patients with ET are needed to exclude from early myelofibrosis, bone marrow examination is still important, and all ET patients had bone marrow reports. But if JAK2V617F or CALR mutation showed positive, even bone marrow examination could not fit the diagnosis, ET is still diagnosed (14). This retrospective study was approved by the Institutional Review Board of Buddhist Tzu Chi General Hospital (Approval No- IRB109-038-B). The need for informed written consent was waived because the study was a retrospective data analysis.

### Predictors

Potential predictors including patient demographics and clinical laboratory parameters, including age, sex, white blood cell (WBC) counts, hemoglobin (Hb) level, mean corpuscular volume (MCV), platelet counts (PLT), mean platelet volume (MPV), platelet distribution width (PDW), and differential WBC count were considered for predicting ET. It is standard practice to perform clinical laboratory tests when patients suffer from thrombocytosis. Thus, these values are readily available. Furthermore, medical records of the patients were assessed to determine the etiology of thrombocytosis.

### Statistical Analysis

Summary statistics including mean with standard deviation and the frequency with the percentage were provided for quantitative variables and qualitative variables, respectively. The Mann-Whitney *U* test for quantitative variables and Chi-square test for qualitative variables were performed to evaluate the differences in subject characteristics between the two groups.

The receiver operating characteristic (ROC) curve analysis was used to decide the optimal cutoff value of each potential predictor, based on the Youden index. The area under curve (AUC), cutoff level, and the corresponding sensitivity and specificity based on the decided cutoff level were calculated for each potential predictor. Multivariate logistic regression with forward model selection method was performed to determine the predictors for predicting ET. In the model, each potential predictor was redefined as a two-level factor based on its cutoff value, obtained from the ROC curve analysis. The Hosmer-Lemeshow goodness-of-fit test was further used to evaluate the calibration ability of the prediction model. Factors with *p* < 0.05 in the multivariate analysis were referred statistically significant and were incorporated into the risk prediction model. The regression coefficient from each factor was transformed to a risk score by dividing its regression coefficients by the smallest regression coefficient among the factors and rounding the quotients to the nearest integer for developing the risk scoring system. The optimal cutoff value of the risk scoring system and the corresponding sensitivity and specificity, based on the optimal cutoff value, were obtained using the ROC curve analysis.

All *p* values were two-sided, with a value < 0.05 considered statistically significant. All data were analyzed using SPSS software (SPSS Inc., Chicago, IL, USA).

## Results

### Characteristics of the Study Patients

A total of 145 patients were enrolled in this study; 66 patients were diagnosed with ET and 79 had RT. The characteristics of the two groups are presented in Table 1. A significant difference between the two groups was observed with respect to age, WBC count, Hb concentration, platelet count, MPV, and PDW (*P* < 0.05).

**Table 1:** Characteristics of patients.

	**Essential thrombocytosis (ET)**	**Reactive thrombocytosis (RT)**	***P***
	***N*** **= 66**	***N*** **= 79**	
Male: Female	35:31	47:32	
Age (years)	63.6 ± 15.9 (59.8–67.5)	55.6 ± 15.6 (52.1–59)	0.003**
WBC (/μL)	16,508 ± 12,267 (13,549–19,468)	11,654 ± 5,920 (10,349–12,959)	0.002**
Hb (g/dL)	12.5 ± 2.37 (11.9–13)	10.4 ± 2.71 (9.76–11)	<0.001**
MCV (fL)	85.5 ± 12.1 (82.6–88.4)	83.9 ± 10.4 (81.6–86.1)	0.384
Platelet (*10^3^/μL)	917 ± 353 (831–1,002)	597 ± 193 (554–639)	<0.001**
MPV (fL)	9.61 ± 0.9 (9.39–9.82)	8.94 ± 0.52 (8.82–9.05)	<0.001**
PDW	11 ± 1.78 (10.6–11.4)	9.49 ± 1.08 (9.25–9.73)	<0.001**
Neutrophil (%)	70.6 ± 12 (67.7–73.5)	67.1 ± 15.8 (63.6–70.6)	0.150
Lymphocyte (%)	17.8 ± 10 (15.4–20.2)	19.8 ± 12.8 (17–22.7)	0.297
Monocyte (%)	5.92 ± 2.68 (5.27–6.57)	6.81 ± 3.78 (5.97–7.64)	0.111
Neutrophil-Lymphocyte ratio	634 ± 554 (501–768)	727 ± 1,176 (468–986)	0.557
Platelet-Lymphocyte ratio	82172.8 ± 77,815 (63,399–100,946)	59518.2 ± 86,547 (40,433–78,603)	0.103

** *P < 0.05*.

*WBC, White blood cell counts; Hb, Hemoglobin; MCV, Mean corpuscular volume; MPV, Mean platelet volume; PDW, Platelet distribution width*.

Among patients with ET, 42 patients (63.7%) had JAK2 V617F mutation, 15 patients (22.7%) had CALR mutation, and 9 patients (13.6%) were negative for both JAK2 V617F and CALR mutations (in our hospital, MPL cannot be assessed). The etiology in patients diagnosed with RT included 19 (24.1%) cases with iron-deficiency anemia without cancer, 34 (43%) with infection, 17 (21.5%) with malignancy, and 9 (11.4%) with other causes.

### Establishment of the Primary Model

Table 2 presents the results of ROC curve analysis for each potential predictor. The AUROC (area under ROC) showed that Hb (AUROC = 0.737), platelet (AUROC = 0.794), MPV (AUROC = 0.753), and PDW (AUROC = 0.785) had high predictive ability (AUROC > 0.7) for predicting ET, while those of MCV (AUROC = 0.544); counts of neutrophils (AUROC = 0.557), lymphocytes (AUROC = 0.539), and monocytes (AUROC = 0.558); and neutrophil-lymphocyte ratio (AUROC = 0.542) were relatively lower (AUROC <0.6). Among the potential predictors, Hb and PDW showed the highest specificity (0.773) and sensitivity (0.886), respectively.

**Table 2:** Results of ROC curve analysis for each positive risk factor.

	**AUC**	**95% CI**	**Cut-off**	**Specificity**	**Sensitivity**
Age (years)	0.637	0.546–0.728	55	0.742	0.519
WBC (/μL)	0.668	0.579–0.757	13,500	0.530	0.772
Hb (g/dL)	0.737	0.655–0.818	10.9	0.773	0.671
MCV (fL)	0.544	0.449–0.64	84.9	0.636	0.481
Platelet (/μL)	0.794	0.719–0.868	659,000	0.758	0.759
MPV (fL)	0.753	0.668–0.837	9.3	0.636	0.861
PDW	0.785	0.707–0.863	10.5	0.621	0.886
Neutrophil %	0.557	0.463–0.652	67	0.697	0.494
Lymphocyte %	0.539	0.444–0.634	14	0.439	0.684
Monocyte %	0.558	0.462–0.653	4.5	0.379	0.810
Neutrophil-Lymphocyte ratio	0.542	0.448–0.637	432.26	0.530	0.595
Platelet-Lymphocyte ratio	0.668	0.568–0.757	38687.5	0.682	0.646

*ROC curve, Receiver operating characteristic curve; AUC, Area under curve; WBC, white blood cell counts; Hb, Hemoglobin; MCV, Mean corpuscular volume; MPV, Mean platelet volume; PDW: Platelet distribution width*.

Multivariate logistic regression analysis demonstrated four independent risk predictors for distinguishing ET and RT based on forward model selection method: WBC (/μL), Hb (g/dL), platelet count (/μL), and MPV (fL) (Table 3). Thus, we adopted these to develop the scoring system to differentiate between ET and RT. The Hosmer-Lemeshow goodness-of-fit test indicates good calibration of this primary predictive model (Chi-square: 5.099; *p* = 0.531).

**Table 3:** The results of multivariate analysis by forward stepwise.

	**Coefficients for each variable**	**Standard error**	***p***	**95% CI**	
				**Lower**	**Upper**
WBC (/μL) >13,500	−1.079	0.527	0.041	0.121	0.955
Hb (g/dL) >10.9	−2.689	0.625	<0.001	0.020	0.231
Platelet count (/μL) >659,000	−3.219	0.629	<0.001	0.012	0.137
MPV (fL) >9.3	−2.226	0.546	<0.001	0.037	0.315

### Establishment of the Scoring System

**Figure 1:**
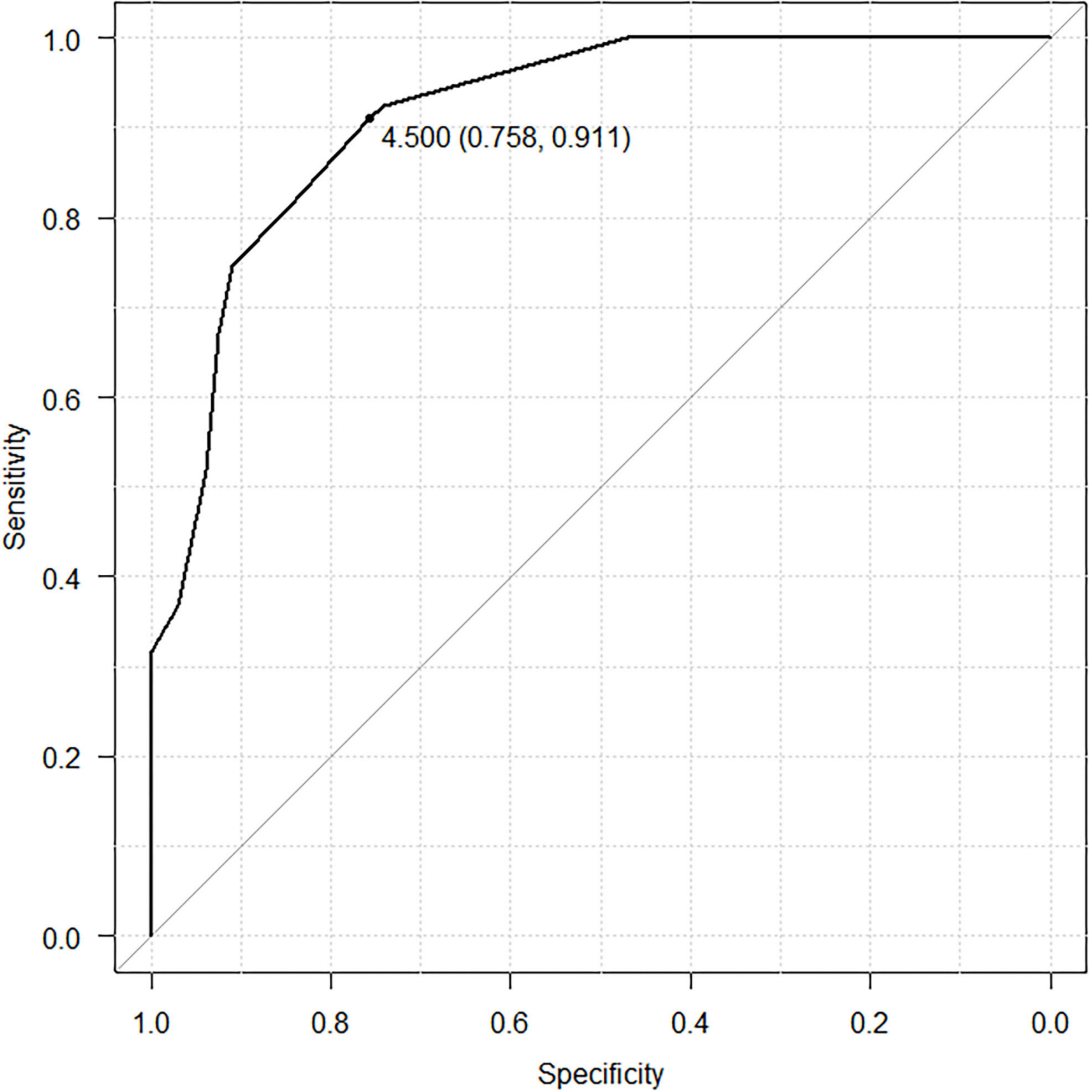
ROC curve of scoring system.

We assigned risk scores relative to the regression coefficient of each variable that showed statistical significance in the multivariate analysis (Table 4): 1 point for WBC > 13,500/μL; 2.5 points for Hb > 10.9 g/dL; 3 points for platelet count > 659,000/μL; and 2 points for MPV > 9.3 fL. In this scoring system, the total score was calculated by adding the individual scores corresponding to the related variables. The prediction accuracy of this scoring system was measured by ROC curve analysis. A sensitivity of 91.1% and a specificity of 75.8% demonstrated the efficient distinguishing ability between ET and RT, achieved by using 4.5 points as the cutoff value (Figure 1), which further indicates that the distinguishing scoring system has made complete use of the primary predictive model.

**Table 4:** The cut-off and points for scoring system.

	**Cutoff**	**Points**
WBC (/μL)	>13,500	1
Hb (g/dL)	>10.9	2.5
Platelet count (/μL)	>659,000	3
MPV (fL)	>9.3	2
Final cutoff by ROC curve		4.5 points

## Discussion

Since thrombocytosis is a common finding in hospitalized patients, distinguishing between ET and RT is important for determining the treatment plan and to ensure a good prognosis. Thus, we have developed a reliable, convenient-to-use, and economical scoring system, comprising four evaluable factors, to distinguish between ET and RT. This model utilizes routine laboratory data for differentiating the two types of thrombocytosis. The simplicity of the scoring system and the high sensitivity are important strengths of our model. This makes it capable of verification by other users, including clinicians, thus enabling its widespread use.

Essential thrombocytosis may result in the development of myelofibrosis and acute leukemic transformation, while RT is generally regarded as benign and has infrequently been reported to cause severe and even fatal complications (15, 16). Many tools and parameters have been used to differentiate ET and RT in the past. Alexandrakis et al. used the levels of serum cytokines and acute-phase proteins to characterize reactive and cancer-related thrombocytosis. A positive correlation was observed between ESR, plasma interleukin-1 alpha, IL-6, and C-reactive protein (17).

Wang et al. attempted to use amount of IGF-1 receptor in the differential diagnosis. The levels of IGF-1R were significantly elevated in ET groups compared to in RT groups or controls (18). Ryningen et al. reported that RT or thrombocytopenia will increase platelet turnover, thus increasing the circulating number of reticulated platelets in myeloproliferative disorders. Ryningen et al. reported that RT or thrombocytopenia will increase platelet turnover, thus increasing the circulating number of reticulated platelets in myeloproliferative disorders. Although RT will increase platelet turnover and the circulating number of reticulated platelets, the conclusion showed reticulated platelets also were noted in hematological malignancies, and circulating reticulated platelets are not available in all hospitals. Circulating reticulated platelets for distinguished ET or RT are not the best methods (19). Circulating thrombopoietin concentrations were also assessed in some studies. Karakuş et al. reported that increased levels of thrombopoietin were found in patients with ET (20). But the tests mentioned in these studies, including assessing for cytokine, IGF-1, and thrombopoietin levels and reticulated platelet count, are not available in all hospitals and are not carried out as part of the routine serum chemistry studies. In addition, these tests are not consistent.

Since the discovery of JAK2V617F, CALR, and MPL mutations as phenotypic driver mutations in essential thrombocytosis (9–11), the diagnostic workup of patients with essential thrombocytosis has been characterized by molecular study (12). However, there is still a proportion of patients who do not exhibit these molecular mutations (13). Triple-negative ET, absence of these driver mutations, may be difficult for differential diagnosis. Although bone marrow biopsy could help to carry out in some cases, it still was difficult to perform gene sequencing to identify clonal marker gene mutations.

Based on previous reports, routine complete blood counts (CBC) could help distinguish ET and RT; additionally, CBC can be easily performed at all clinics and hospitals. Sehayek et al. used the platelet parameters and aggregation to compare essential with reactive thrombocytosis. The results showed that MPV was significantly lower in patients with ET vs. those with RT. A platelet volume distribution width ≥10.5 was found in 50 and 21% of ET and RT patients, respectively. But the authors had only enrolled 15 patients with ET (21). Michael G Alexandrakis et al. reported that the levels of hemoglobin, hematocrit, and platelets were significantly lower in RT than in ET (17). Toprak et al. in their study, showed MPV, hemoglobin, red blood cell indices, white blood cell counts, serum iron profile, and C-reactive protein levels were helpful in the differential diagnosis of thrombocytosis. Their study comprised 49 patients with RT and ET. The MPV level in RT group was less than that in the ET group (7.49 and 8.80 fL, respectively, *P* < 0.01) and had a sensitivity of 65% and specificity of 89% (22). In our study, we used the routine data of CBC and differential count of white blood cells for designing the scoring system. After analysis by SPSS, four independent risk factors were included in the system: (1) white blood counts, (2) hemoglobin level, (3) platelet counts and (4) mean platelet volume. The cutoff levels were set as: WBC > 13,500/μL, Hb > 10.9 g/dL, platelet count > 659,000/μL, and MPV > 9.3 fL. The scoring system was created by assigning 1 point for WBC, 2.5 points for Hb, 3 points for platelet counts, and 2 points for MPV. The cutoff value was a score of 4.5. If the score was more than 4.5 points, diagnosis of ET was made, with a sensitivity of 91.1% and a specificity of 75.8%.

Although only a few patients who might miss the diagnosis of ET, the risk of thrombotic events, marrow fibrosis, and acute leukemia transformation were still needed to consider. Therefore, distinguishing between the two types is essential for clinical practice. For the purpose of this study, we hope to design the scoring system for “screening.” High sensitivity and convenience to use are more important than specificity.

This study still has several limitations. First, an inherent selection bias due to the retrospective study design cannot be avoid. And we only enrolled the ET patients who was diagnosed with bone marrow examination and molecular study by hematologists. The RT patients were enrolled with thrombocytosis once and then recovery of platelet count later. If the patients did not refer to the hematologic department, they had been excluded from this study. An overt selection bias was noted. Although above, we hope to design the scoring system for the doctors of family medicine or general practitioners, except hematologists and help them to aware of ET. Second, the sample size was still small although we have collected more cases than previous submission. A further prospective cohort with a larger sample size is needed to validate our screening scoring system.

## Conclusion

In this study, we used just one routine lab data to build a high-sensitivity scoring system to screen the patients with thrombocytosis to possible ET patients and possible RT ones. The four factors were white blood counts, hemoglobin level, platelet counts, and MPV. The scoring system was assigned to the resulting model to make it more economical, simple, and convenient for clinical practice. We hope to design a scoring system for family medicine or general practitioners to identify possible bone marrow disease and consult hematologists, and then transfer the patients to hematologists for further bone marrow examination and molecular studies. If the patients were found and transferred to hematologists, further treatment, including cytoreduction, prevention from thrombosis, evaluation about myelofibrosis or leukemic transformation, will be greatly helpful for these patients.

## Data Availability Statement

The data analyzed in this study is subject to the following licenses/restrictions: Due to IRB, raw data were generated at Hualien Tzu Chi Hospital. Derived data supporting the findings of this study are available from the corresponding author Yi-Feng Wu on request. Requests to access these datasets should be directed to Yi-Feng Wu, wuyifeng43@gmail.com.

## Ethics Statement

The studies involving human participants were reviewed and approved by Institutional Review Board of Buddhist Tzu Chi General Hospital (Approval No. IRB109-038-B). Written informed consent for participation was not required for this study in accordance with the national legislation and the institutional requirements.

## Author Contributions

C-LS, T-CH, and Y-FW contributed to data analysis and manuscript editing. T-FW, W-HH, and S-CC collected the data of patients. C-LS and T-CH helped in manuscript preparation. Y-FW contributed to the supervision of the whole process. All authors contributed to the article and approved the submitted version. The work reported in the above for publications has been done by all authors.

## Conflict of Interest

The authors declare that the research was conducted in the absence of any commercial or financial relationships that could be construed as a potential conflict of interest.

## Publisher's Note

All claims expressed in this article are solely those of the authors and do not necessarily represent those of their affiliated organizations, or those of the publisher, the editors and the reviewers. Any product that may be evaluated in this article, or claim that may be made by its manufacturer, is not guaranteed or endorsed by the publisher.
